# Models in Pancreatic Neuroendocrine Neoplasms: Current Perspectives and Future Directions

**DOI:** 10.3390/cancers15153756

**Published:** 2023-07-25

**Authors:** Steven D. Forsythe, Tracey Pu, Stephen G. Andrews, James P. Madigan, Samira M. Sadowski

**Affiliations:** 1 Neuroendocrine Cancer Therapy Section, Surgical Oncology Program, National Cancer Institute, National Institutes of Health, Bethesda, MD 20892, USA; steven.forsythe@nih.gov (S.D.F.); stephen.andrews2@nih.gov (S.G.A.); james.madigan@nih.gov (J.P.M.); 2 Surgical Oncology Program, National Cancer Institute, National Institutes of Health, Bethesda, MD 20892, USA; tracey.pu@nih.gov

**Keywords:** pancreatic neuroendocrine neoplasms, models, cell lines, mouse models, 3D cell culture

## Abstract

**Simple Summary:**

Pancreatic neuroendocrine neoplasms (pNENs) are a rare and understudied cancer. Some of this low knowledge base is due to a historical lack of study models. Study models have tremendous implications for validating data for a range of cancer research topics, including treatment development. Therefore, correctly choosing a model is imperative and needs to consider a range of factors pertinent to the research question. In this review, we summarized the current field of models in pNENs. We considered factors, including complexity, accuracy, and cost, in models ranging from cell line cultures, 3D cultures, and whole organismal models, including mice and zebrafish. Improving the number and quality of models available will lead to new breakthroughs in treating pNENs and may lead to findings beneficial for other cancers.

**Abstract:**

Pancreatic neuroendocrine neoplasms (pNENs) are a heterogeneous group of tumors derived from multiple neuroendocrine origin cell subtypes. Incidence rates for pNENs have steadily risen over the last decade, and outcomes continue to vary widely due to inability to properly screen. These tumors encompass a wide range of functional and non-functional subtypes, with their rarity and slow growth making therapeutic development difficult as most clinically used therapeutics are derived from retrospective analyses. Improved molecular understanding of these cancers has increased our knowledge of the tumor biology for pNENs. Despite these advances in our understanding of pNENs, there remains a dearth of models for further investigation. In this review, we will cover the current field of pNEN models, which include established cell lines, animal models such as mice and zebrafish, and three-dimensional (3D) cell models, and compare their uses in modeling various disease aspects. While no study model is a complete representation of pNEN biology, each has advantages which allow for new scientific understanding of these rare tumors. Future efforts and advancements in technology will continue to create new options in modeling these cancers.

## 1. Introduction

Pancreatic neuroendocrine neoplasms (pNENs) are a rare class of tumors which derive from neuroendocrine cells located in the islets of the pancreas. They are the third most common neuroendocrine tumor subtype in the gastro–entero–pancreatic (GEP) system, accounting for 1.2 cases per 100,000 people and showing a steady rise over the last few decades globally [1]. The reasons for increased incidences are not fully understood; however, it is likely in part due to increased surveillance and awareness. Five-year survival for pNENs is at 37.6%, significantly higher than pancreatic adenocarcinoma (PDAC) at <5% despite being less than 2% of total pancreas tumors [2]. One of the reasons behind this enhanced survival is the relative indolence of these tumors. Many pNENs grow slowly and have delayed metastatic capability, extending patient survival. However, this is counteracted by the lack of early disease indications, and the long latency between initiation and discovery leading to medical complications and the presence of metastatic lesions upon diagnosis.

Classification of pNENs falls into several phenotypic and genomic categories [3]. Grading of the tumors can be broken into categories based on ki67 proliferative index and cell differentiation. Currently, there are three grades of pNENs: pancreatic neuroendocrine tumors (pNETs) of Grade 1 (G1, well differentiated (WD), ki67 < 2%), Grade 2 (G2, WD, ki67 between 2–20%), and Grade 3 (G3, WD, Ki67 > 20%) [4]. In G3, there is further delineation from WD into poorly differentiated (PD), also known as pancreatic neuroendocrine carcinoma (pNEC) [5]. The difference between these two is defined by a loss of phenotypic identity and change in mutational patterns, characterized by more aggressive tumor behavior in pNECs, and thus they are treated differently. pNECs can also be broken into small and large cell carcinomas, categorized by visible cytoplasmic content and nucleoli; despite this, they are currently treated the same [5]. Grading is persistent between all pNEN subtypes.

**Figure 1:**
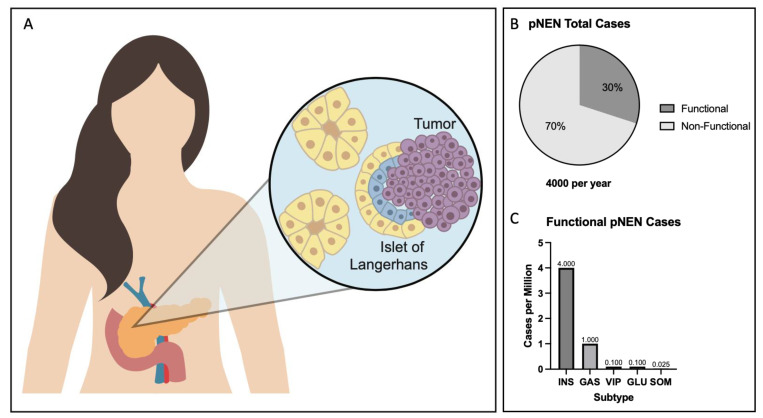
(**A**) Biology of pNEN development. PNENs are theorized to originate from neuroendocrine progenitor cells in the islets of the pancreas, which can take on the characteristics of several islet cell subtypes in functional pNENs. (**B**) Distribution of functional and non-functional pNENs and (**C**) incidence of functional pNEN subtypes. INS—insulinoma, GAS—gastrinoma, VIP—VIPoma, GLU—glucagonoma, SOM—somatinostatinoma.

pNENs comprise a heterogeneous background of subtypes (Figure 1). There are two major classes of pNENs: functional and non-functional tumors. Functional tumors comprise 30–40% of all pNENs and contain five clinical subtypes, in addition to several extremely rare syndromes [6]. They are defined by the creation of hormonal medical issues and are often the reason patients arrive in the hospital. Typically, they have earlier onset than non-functional tumors, with presentation ranging from 40 to 60 years of age. Functional pNENs can be categorized by the cell types they derive from and the hormones they secrete. The pancreatic islet consists of several cell types with unique hormone regulation roles in the pancreas; they are the alpha, beta, delta, pancreatic polypeptide (PP), ghrelin, and gastrin cells [7]. Insulinomas are the most common functional pNEN (~4 per million individuals) and are derived from beta cells in the islets. They overproduce insulin, which can cause hypoglycemic symptoms in patients, leading frequently to severe illness. They are often benign, and curative outcomes are possible with appropriate treatment. Gastrinomas are the next most common functional pNEN (~1 per million) and derive from progenitor islet alpha and beta cells [8]. They overproduce gastrin, which can lead to Zollinger–Ellison syndrome, creating ulcers, diarrhea, and gastric reflux disease. They are heavily influenced by genetics with a typically earlier onset with a high risk of metastases. VIPomas (~0.1 per million) are associated with Verner–Morrison syndrome and are derived from non-beta cells. They oversecrete vasoactive intestinal peptide (VIP), which causes severe diarrhea and hypokalemia. They are aggressive, of early onset and characterized by single-lesion tumors with a lower genetic component than other pNEN subtypes. Glucagonomas (0.1 per million) are derived from the alpha cells of the islet and present as large tumors. Over-secretion of glucagon leads to a variety of symptoms, including onset of diabetes mellites, characteristic rashes, and weight loss. Onset is early (30–50 years of age), and there are heavy genetic components involved with development. Finally, somatostatinomas (0.025 per million) are extremely rare tumors derived from the delta cells of the pancreatic islet. They overproduce somatostatin and present as a single large lesion, clinically leading to diabetes and gallstones. In addition to the five described above, there are a few extremely rare pNEN subtypes, which include the ACTHoma, GRFoma, pNENs causing carcinoid syndrome, and PTHrPomas [9]. Non-functional (NF) tumors encompass the remaining 60–70% of pNENs and do not produce overt symptoms [10]. They are slow growing, late presenting tumors which are usually detected during examinations for other medical issues as the symptoms for NFs are typically non-specific. Despite their non-functional label, they do secrete markers which may help in detection, including chromogranin-A, ghrelin, PP, neurotensin, motilin, neuron-specific enolase, or human gonadotropin. Overall, there is significant tumor subtype diversity requiring special consideration for treatments.

The genomic characterization of pNENs from initiation to cancer has only been covered in the recent literature [11,12]. Despite encompassing a significant number of subtypes with unique behavior, pNETs carry relatively few mutations, which are often limited to tumor-suppressing functions [12]. Roughly 10% of cases are related to familial endocrine tumor syndromes and include multiple endocrine neoplasia type 1 (MEN1), von-Hippel Lindau disease (VHL), neurofibromatosis type 1 (NF-1), and tuberous sclerosis (TSC). There are four major functions affected in pNENs: DNA damage repair, chromatin modification, telomere length alterations, and mTOR signaling. *MEN1* is the most common altered gene and is found mutated in 40–50% of pNEN patients and is associated with both a germline condition causing numerous endocrine tumors as well as with sporadic biallelic inactivation [11]. *MEN1* is involved in a wide range of pathways, including regulation of DNA transcription and replication, maintenance of genome integrity, and control of cell cycle, and it is believed mutations in *MEN1* can prevent the nuclear localization of menin, the protein encoded by the MEN1 gene, losing these important functions [13]. There are two major mutations to genes involved in telomere lengthening: *DAXX* (22–25%) and *ATRX* (10–18%) [12,14]. *DAXX* has a wide range of interactions, including cell cycle and apoptosis control, while *ATRX* is involved in chromatin remodeling during the cell cycle [15]. Control of telomere lengthening ensures cells do not divide indefinitely, and loss of this ability causes an increase in genetic anomalies and loss of apoptotic control. These two genes are found mutated mutually exclusive to each other in patients, and their presence can assist in determining tumor grade. Mutations to the PI3K-AKT-mTOR signaling pathway have been described in several studies [12]. These mutations are involved in both familial syndromes (TSC and Cowden’s syndrome) and in somatic mutations and each typically regulates activation of mTOR pathways family member proteins. *PTEN* loss is found in 7% of all cases [16]. It prevents the activation of AKT by PI3K signaling, and loss of PTEN function increases oncogenic signaling through the PI3K-AKT-mTOR pathway. *TSC* members 1 and 2 are found to be mutated in a small percentage of cases (2% each); however, they form an autosomal dominant phenotype (*TSC* syndrome) as the loss of one copy is sufficient for tumorigenesis. TSC1 (hamartin protein) and TSC2 (tuberin protein) negatively regulate activation of mTORC1 through inhibition of the small GTPase Rheb, a positive regulator of mTORC1. Finally, DEPDC5, a Rag inactivating protein, is observed to have biallelic inactivation in 2% of cases. Mutations to DNA damage repair proteins have a threefold effect on tumorigenesis; they promote further genomic instability, prevent checkpoint activation, and increase treatment resistance. MUTYH, a protein involved in base excision repair, was found to have both germline mutations and biallelic inactivation (5%) in patient specimens. Mutations to checkpoint kinase 2 (CHK2), regulator of the G1-S phase transition, have also been observed in a subset of patients (2% biallelic inactivation). Finally, mutations to chromatin-modifying proteins, including histone methyltransferases SETD2 (4% biallelic) and MLL3 (5%), are observed in patients. Alterations to INK4A/ARF locus, which encodes p16^INK4a^ and p14^ARF^, can also limit the expression of tumor suppressor *Rb1* and *p53* by failing to block cell cycle activating proteins. Evidence has shown that although these genes are not often mutated in WD pNETs, hypermethylation may cause inactivation of these genes, in particular for gastrinomas [17,18]. In fact, recent research has suggested epigenetic expression of genes may have an outsized role in pNEN development, and studies have recommended using therapeutics including HDAC inhibitors, such as entinostat, to target these tumors [19,20]. In contrast to pNETs, pNECs carry more aggressive mutational profiles, with activation of oncogenes KRAS, CDK4/6, SMAD4, and Bcl-2 at a high rate [5,21]. Similarly, these tumors often lose expression of *Rb1* and *p53*, demonstrating loss of tumor suppression in addition to the oncogenic gains [22]. As technology continues to improve, including single-cell analysis, new insights will further elucidate the evolution of pNENs.

Treatments of pNENs remain limited. The current gold standard and only curative treatment is surgical removal [23]. In most localized pNEN tumor subtypes, this will be sufficient to eliminate the tumor and relieve patients of overt hormonal emergency. Due to tumor indolence, there has been debate on the necessity of surgery while patients are asymptomatic; in general, tumors of <2 cm with no symptoms are given surveillance [10]. Depending on tumor location and tumor stage, surgical procedures include enucleation for small tumors in the pancreatic head, distal pancreatectomy for body or tail tumors, pancreaticoduodenectomy for head tumors incapable of enucleation, or total pancreatectomy. If disease is advanced or metastatic, further operative treatment should be considered, including ablation of liver metastases. Hormone control therapy for functional pNENs is required to improve patient comfort and to avoid life threatening complications. For controlling tumor growth, several classes of therapeutics have been clinically utilized. Somatostatin receptors (SSTRs) are found upregulated in a variety of pNENs, making both imaging and therapeutic targeting technologies a promising therapeutic avenue [24]. Targeted therapy using somatostatin class mimics for SSTR2 to control hormone production is a first-line medicine treatment for most subtypes, with insulinomas excepted [25,26]. Other targeted therapies have also been investigated, with sunitinib and everolimus the two most investigated therapies targeting VEGF and mTOR, respectively [27]. The usage of chemotherapy has limited success, with a wide range of single agents and regimens used in practice. Treatments including streptozotocin, oxaliplatin, and dacarbazine/temozolomide have been used as single agents or in combinational regimens. However, due to the slow growth of pNENs, the criteria for initiating treatment has been controversial, with important factors including ki67 score and tumor subtype needing consideration [28]. Usage of radiation through targeted radiotherapy has been a growing treatment consideration in pNENs. Conjugated 177 lutetium-dotatate with SSTR2 targeted treatments have shown promising results in improving patient outcomes in a variety of studies with pNENs [29,30]. A variety of other treatments, including further targeted therapies, epigenetic modifying agents, and immunotherapies are currently in clinical trials [23,27,31,32]. Despite these advances, further work is needed to transition the treatments of pNENs from generalized therapies towards a more precision medicine approach.

**Figure 2:**
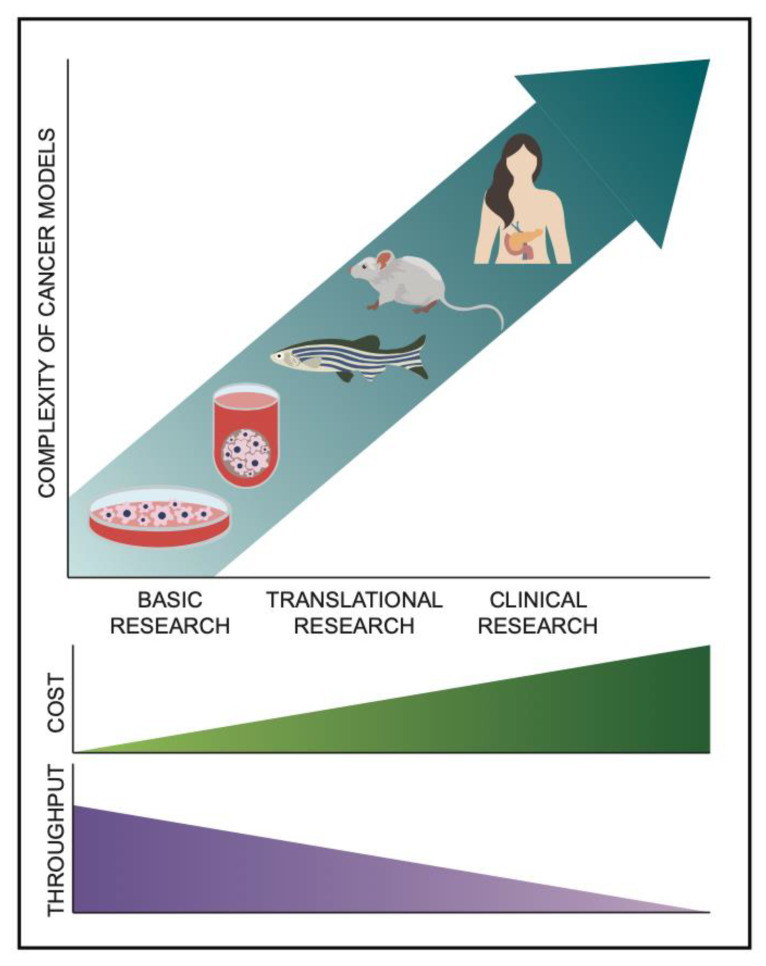
A comparison of models of pancreatic neuroendocrine neoplasms comparing the complexity, ease of reproducibility (throughput), and cost of several available options, including immortalized cell lines, three dimensional primary derived cells, zebrafish, mice, and humans.

To better understand pNENs from initiation, progression, and treatment, proper study models are required. Choosing the correct model is imperative to create an accurate representation of this cancer (Figure 2). Each model contains strengths and weaknesses which must be considered when used in research. In this review, each of the models will be described, with the available research provided on their utility in studying pNENs. The major groups of models covered are cell line models, animal models, and three-dimensional cellular models. 

## 2. Cell Lines of pNENs

In scientific research, there is often a need to create large scale, high-throughput experiments to address a variety of hypotheses. Cell lines have allowed for fast, reproducible results for tissue types throughout the entire body. Isolation of the tumor cell populations often requires specialized culture techniques including substrate and cell culture medium selection. They are a useful model for cancer as the process to create stable, “immortalized” cultures can often mimic the development of tumors. There are often mutations in tumor suppressor genes, including p53, and they may have alterations to hTERT, conferring the long-term ability to reproduce without cell forced senescence or apoptosis, also known as the Hayflick limit [33,34]. These alterations are present either through acquired mutations before long-term culture or are induced by researchers, typically through viral oncogenic transformation [35]. Due to the consistency of cell line models over time, complex experiments, including genetic alteration to create multiple subclonal populations with features of interest, such as lentiviral transfection or CRISPR/Cas9, are possible [36]. pNEN cell lines have been under development since the 1970s from a variety of sources, including both animals and humans. Herein, we describe the current literature on pNEN cell lines (Table 1).

### 2.1. Animal-Derived Cell Lines

Cell lines derived from animals are advantageous as animal models have less stringent requirements when compared to human studies for cellular acquisition. They can be generated from parent organisms through indirect genetic manipulation, such as radiation bombardment or by targeted viral infection to induce oncogenesis. Many of the animal pNEN cell lines are derived from early-stage insulinomas and were developed to create cell models for diabetes research, making stable long-term insulin-producing and responding lines [58]. Oncogenic transformation is often performed with an oncogenic virus directly induced into pancreas islets, which causes tumor-like alterations to *p53* and Rb1, as seen in pNECs [59]. It is also common for animal cell lines to have isolated daughter clonal populations to select for various traits. As the field is predominantly insulinomas, there remains lower coverage for the other subtypes of pNETs as compared to human cell lines, especially in context to more aggressive tumors.

The most common host species for animal cell lines are mice. Mouse models for pNENs have been extensively developed, and work to establish cell lines for study has persisted for some time. Early work included attempts to create cell lines isolated from beta cells in insulinoma-like pancreas islets for the study of diabetes using the SV40 large T antigen. These cell lines, including βTC, MIN6, BTC-5, NIT-1, and TCP61, have been utilized in a wide range of research for prediabetes and for the study on early stage insulinomas [37,38,40,42,43]. There have also been efforts to model glucagonoma tumors by isolating affected alpha cells in the islets. The alpha TC1 cell line, isolated from preproglucagon promoter SV40-T-antigen-induced alpha cells in islets, has been extensively utilized in research involving glucagon secretion [39]. It has been well characterized and compared to beta cells to create a distinct network of genomic profiles [60]. Another potential glucagonoma cell line, Mu Islet (E6/E7), was developed by ATCC using human papillomavirus (HPV) type 16 with proteins E6 and E7, the first using this transformation for pNEN cell lines [44]. Although this line is available, there is no literature on its use in research. 

There are currently four cell line models of pNENs from animal species other than mice. Two rat cell line models, RIN and INS1, were derived from the same rat line, the inbred NEDH (New England Deaconess Hospital) rat [45,46,61]. The cell lines were isolated independently from rats which had undergone X-ray irradiation to induce insulinomas. The RIN1 cell line has been utilized extensively and several “daughter”-derived cell lines are currently available which have been selected for various secretory differences [62,63]. INS1s have been studied for their insulin secretion stability and also contain several daughter clonal isolate populations to further enhance this understanding [64]. The HIT cell line was isolated from the SV40-T-antigen-transformed islets of a Syrian hamster [47]. It has been extensively analyzed in glucose and insulin sensitivity testing in a wide range of research applications [65,66]. Finally, the canINS cell line was isolated from a dog with an insulinoma and has been noted for its ability to maintain cancer stem cell (CSC) populations and insulin secretion, although this is limited to certain culture methods [48]. Overall, while there are several animal cell lines available, the amount of cancer-related research is limited.

### 2.2. Established Human Cell Lines 

Efforts to establish pNEN cell lines from human tumors has been an important experimental objective. It involves taking cells from primary or metastatic tissues to create long-term study models to mimic the disease process while providing a more biologically relevant model when compared to animal cell lines. This approach is not without its pitfalls; while the mimicry of the disease state is important, slow growth characteristics of pNENs can mean poor establishment success along with long cell-doubling times, which makes therapeutic testing difficult [52,55]. Changes in cell line characteristics such as hormone secretion and genetic drift have also been noted, leading to questions about their utility [57,67,68]. Because of this, most cell line models are derived from either G3 tumors or pNECs, which limits studies to the most aggressive tumors and not early-stage tumors.

There has been considerable subtype diversity in human-generated cell lines. The earliest published human cell line, QGP-1, was derived from an aggressive somatostatinoma which also produces carcinoembryonic antigen (CEA) [49]. This cell line has been extensively characterized and analyzed in many therapeutic sensitivity studies [68,69,70]. BON-1 is the other most characterized cell line and is a well-studied cell line of pNECs, with several oncogenic gain-of-function mutations which make for a valuable study model, including Ras, BRCA2, and TP53 [51]. Another cell line derived from an insulinoma, CM, was derived from patient ascites and has been used for over 40 years, with positive data comparing its insulin secretion to cells, although these results have been the subject of controversy in more recent studies [50,67,71]. A more recent insulinoma cell line from a WD tumor, NT-3, has shown promise as an accurate representation of mature beta cells, and has already drawn extensive preclinical research with SSTR2 treatments [55,68,72]. One cell line for pancreatic small cell carcinoma, A99, has been described in the literature, which contains point mutations at KRAS (G12L) and TP53 (S127T), along with comprehensive chromosomal rearrangement errors which has made it treatment resistant [53,73]. A cell line representing VIPoma, HuNET, has been isolated using a collagen IV coated transwell plate and demonstrated VIP secretion, although it has been observed to decrease with increased passages in culture [52]. Efforts to create cell lines from grade 1 tumors have recently been successful with the creation of the APL1 cell line, which maintained the low rate of division by measure of Ki67 activity [54]. This cell line was tested with CD47 monoclonal antibody therapy combined with EGFR inhibitors to provide significant treatment efficacy. Recently, a series of cell lines was generated from the same patient during their cancer progression [57]. These cell lines, NT18P (primary), NT-18LM (lymph node metastasis), and NT-36 (primary recurrence), allowed for researchers to study the clonal evolution of a tumor cell population, with the authors noting the development of a mutation in DAXX for the NT-18LM line that allowed for tyrosine-kinase-inhibitor (TKI) targeting. The authors also were able to create a cell line from a large cell pNEC, NT-32, which demonstrated a targetable mutation in BRAF. Finally, SPNE1, a cell line from a nonfunctional primary pNET, has shown promise with a high percentage of CD44/CD117+ cancer stem cells when compared to other available pNEN cell lines and was extensively characterized with whole-exome sequencing [56].

### 2.3. Primary Human 2D Cultures 

In addition to the long-term cultured cells described above, there have been recent efforts to culture primary cells taken by biopsy for experimental study. The goal for doing so is to more accurately model pNENs by controlling the potential of genetic drift in long-term culture. A comparison of treatment combinations involving everolimus and somatostatin analogues performed using tumor cultures of primary pNET cells cultured with bovine ECM has been described by Mohamed et al. [74,75]. Many of the tumors were non-functional tumors, with insulinoma and gastrinoma also reported. The studies concluded while differing SSTR2 targeting therapies had differing effects on SSTR2 internalization, the combinational therapy did not create a treatment benefit on cell viability when compared to each therapeutic alone. Another study was performed on tumors from 16 patients, with 13 NF and 3 insulinoma tumors by seeding them at low numbers into well plates [76]. The cultures demonstrated both responsive (R) and non-responsive (NR) treatment results to everolimus, with one NR cell culture matching the treatment outcome of its parent patient. The authors also showed the importance of mTOR activity in predicting potential therapeutic response in pAKT positive cells. Although these studies show positive results, the rarity of pNET subtypes and the technical issues of primary cell culture may make propagation and reproducibility a challenge. 

## 3. Animal Models of PNENs 

In modeling cancer, animal models are important to study the whole-body effects of cancer. As entire organisms, these models maintain the tumor and non-tumor components, including stromal, immune, and non-cellular elements, organized into tissues and organs, allowing for important crosstalk between functional systems [77]. Animals can be tested for their ability to form tumors either through carcinogenic exposure or through direct genomic manipulation. This allows for not only the study of the individual tumor types but throughout the body as they invade other tissues. Treatments in animal models are seen as closer to humans than simple models and are important in clinical trial development. Importantly, other model systems, including cell lines and 3D models, can be incorporated into organisms to test their tumor forming abilities. The field on organism modeling in pNENs can be divided into two major groups: xenograft models and genetically engineered organisms, which we will describe here.

### 3.1. Patient-Derived Xenografts of PNENs

Xenograft models of cancer use animals to propagate tumor cells over the course of multiple passages. They are one of the oldest methods for the culture of tumor cells, with studies reported for over 50 years [78]. They can use cells from any source, including established cell lines, animal tumors, or human tissues. Typically, the host for xenografts needs to be immunocompromised by way of non-functional immune cell populations to eliminate the risk of graft rejection. A variety of organisms have been created to fill this need, mostly in mice, including the athymic nude and severe combined immunodeficient (SCID) mice [79]. The methods of tumor cell introduction include heterotopic (different organ site from tumor location) implantation, orthotopic (same organ site) implantation, or by injecting cells into the bloodstream for colonization [79]. pNEN cases typically fall either as a part of large efforts to model neuroendocrine tumors for all pancreatic subtypes or for a few selected pNEN specimens. In this section, we will cover sources from human tissues, also known as patient-derived xenografts (PDXs) (Table 2).

The first published attempt to form pNEN PDX models was reported in non-obese diabetic (NOD)-SCID subcutaneously flank implanted mice during the attempt to create stable lines from several NEN tumors [80]. The authors were able to successfully passage 3/58 pNET tissues in mice once; however, any further passages failed. The first successful effort in generating a long-term PNEN PDX model was performed on an insulinoma into athymic nude mice [81]. A patient tumor with MEN1, BRCA2, PTEN, and SETD2 mutations was passaged in PDX models to create everolimus resistant tumors, followed by treatment with sapaniseritinb, a potent dual mTORC1/2 inhibitor, to overcome resistance. The next study used a new source, organoids, derived from two patients with a pNETG3 and a large-cell pNEC [82]. These were implanted into the renal tubules and showed successful implantation but demonstrated lower proliferation than the original tumor or the PTOs. Another successful attempt was reported in 2020 using subcutaneous implantation in NOD-SCID mice for 1/5 tested tumors [83]. However, as the focus of the paper was on PDAC PDX establishment, little follow-up was performed. The most recent study in mice was incapable of successful implantation with tumor cell subcutaneous injections with pNETG2 tumors [84]. While most PDX efforts have been in mice, there has been one report of PDX modeling in zebrafish. Zebrafish embryos do not have fully developed immune systems and have translucent skin, allowing for easy observation of tumor growth. Gaudenzi et al. used cells from a pNETG1 liver metastasis and injected 100 cells into zebrafish embryos [85]. The tumor cells showed growth and migration over five days and demonstrated evidence of tumor angiogenesis. In brief, while PDX models are a new development in pNEN models, recent efforts have provided the framework for pNEN research.

### 3.2. Genetically Engineered Mouse Models (GEMMs) of PNENs

**Figure 3:**
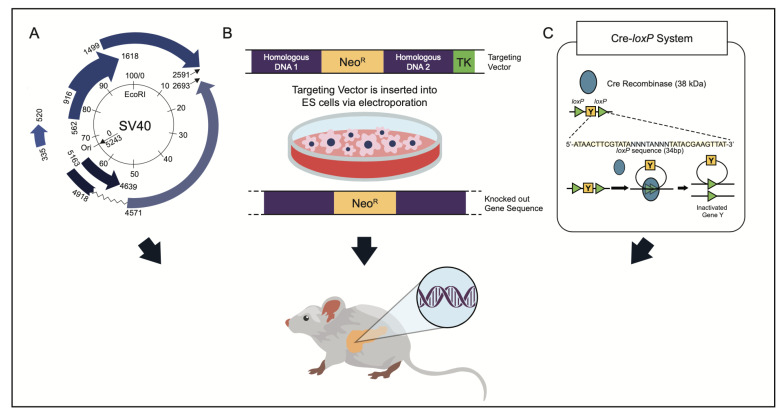
Illustration of commonly used methods of establishing genetically engineered mouse models, including (**A**) SV40 Tag, (**B**) removal or amplification of target gene, and (**C**) Cre-LoxP-induced transformations.

The most developed model system of pNENs is the genetically engineered mouse model (GEMM). Mouse models have been a valuable tool in scientific research for decades; indeed, there may be upwards of thousands of mouse strains developed for research [86]. Early work to create crossbreeds created new understanding of the spontaneously developing phenotypes of many medical conditions and improved the understanding of husbandry. More recently, they allow for genetic manipulation to create disease specific phenotypes and have allowed further knowledge of human cancers. These methods of manipulation have become honed over time, with increasingly targeted methods created with little to no off-target effects (Figure 3). In general, methods of genetic modification can be broken into two categories: those affecting the suppression of the tumor phenotype and those driving oncogenic activity. 

### 3.3. SV40 Tag

One of the earliest methods of induced oncogenesis in mouse models is using oncogenic viruses. The introduction of virus into a target sequence is followed by injection into embryonic cell lines. These cells are then introduced to embryos and can be bred to form stable populations. Commonly, the simian virus 40 large T-antigen (SV40 Tag) sequence is altered to remove the ability for viral reproduction before being incorporated downstream of a tissue-specific promoter of interest. This gene of interest should be “targeted” to the organ-specific function of the cells in question, as limiting the number of cells expressing the SV40 Tag is important in eliminating off-target effects. The promoter of the tissue-specific gene will drive transcription in specific cells which allows for the expression of the SV40 large T-antigen, causing oncogenesis. Mutations which arise from this process often included *p53* or *Rb1*, which are common pNEC genes. 

Of the major developed hybrid target sequences of SV40 Tag, the Rat Insulin Promoter (RIP) model has the most extensive publication record. First described in 1985, the RIP-Tag mouse lines were developed by incorporating the SV40 Tag downstream of the rat insulin promoter (RIP) to create a pancreatic islet beta-cell only transcription sequence [87]. The resulting mice have short lifespans, with completely penetrant hyperplasia of the islets occurring at 8–12 weeks and death occurring within 20 weeks due to severe hypoglycemia by way of arising insulinomas. Rate of metastases in these mice appears to be low, with only modest liver and lymph node metastasis observed and the occurrence of other tumor types is rare. Of the developed RIP-Tag strains, the most utilized are the RIP1-Tag2 (RT2), and RIP1-Tag5 (RT5) (Table 3). RT2 has been used in the most studies and has been crossed extensively with other mice, leading to several novel study models, including bioluminescent beta cells [88]. These breeding crosses involve other oncogenes and can alter tumor phenotype and behavior dramatically. For example, RT2 crossed with RIP mice expressing higher IGF-1R, VEGF-A, VEGF-C, VEGF-D, tenascin-C, or heparinase have been observed to increase tumor growth, angiogenesis, and/or invasion in separate studies [89,90,91,92,93,94]. Interestingly, in a model of Rag^−/−^ adaptive-immunity-depleted mice crossed with RT2, anti-angiogenic treatment would lead to smaller tumors more capable of metastases [95]. In contrast, the loss of E-Cadherin or platelet-PDGFB reduces tumor progression and development [96,97]. Of note, the role of mouse strain on RT2 tumor formation has also been monitored, and the strain of mouse can either alter the rate of tumor formation or favor other pNEN subtypes, including creating NF pNENs, dependent on expression of genes including Insm1 and IGF2 [98,99]. RT5 is notable as several T-antigen studies have been performed, making it a candidate for studies of the immune system [100,101]. RIP-Tag mouse models continue to be used today, with several important efforts to further understand tumor development and behavior. 

There have also been other SV40 target hybrid sequences utilized for the study of pNEN development (Table 4). Based on the target sequence chosen, it is possible, and sometimes expected, for other tumor types to form in addition to pNENs. The time to tumor development varies, with the shortest being 8 weeks and the longest 9–12 months. Examples of these include vasopressin, elastase, metallothionein 1, L-Type pyruvate kinase, gastrin, and secretin, which have all shown pNEN development in addition to a wide variety of other tumor types [104,105,106,107,108]. One group even incorporated the murine sarcoma virus (MSV) sequence with SV40, developing a wide range of tumors including insulinomas [106]. Of particular interest, there have been efforts to target the glucagon sequence, which is highly expressed in alpha cells and results in the development of glucagonomas [109]. However, another group reported the development of colon tumors in a similar model, confirming the targeting of glucagon is not a pancreas specific process [110]. As technologies improved over time and allowed for more direct control over mutagenesis, researchers use more targeted methods of tumor development than these sequences.

### 3.4. Conventional Germline Heterozygous Knockouts

In modeling cancer development, the use of global heterogenous knockouts (KOs), also known as conventional KO models, have found a niche in many organisms [116]. The KOs are often created by removing single copy of the gene of interest and breeding mice colonies to maintain the line. They allow for a more natural tumor growth process which better mimics the process in humans and can demonstrate value in analyzing other linked genomic alterations in the development of tumors. Genes of interest are usually tumor suppressors, including *p53*, *Rb1*, and *MEN1*. They are also useful for modeling genes which cannot experience germline knockouts, such as *MEN1* [117]. If the gene is not dominant, they will require a second inactivating somatic mutation in the other copy, which makes the development of tumors longer and more variable than seen in other model types. Additionally, since a copy of the gene is knocked out throughout the entire mouse, other cancers can form and can cause death before pNENs develop. The most common example of global heterogenous KOs include models of *MEN1* mice [117,118] (Table 5). Studies have determined a rate of roughly 50–60% pNEN formation in mice with this genotype, with a time of development at least 8 months. *Rb1* has also been studied in conjunction with other inactivated mutations, and although *Rb1* heterogenous KOs can form pNENs, work has shown it to not augment tumorigenesis in *MEN1* KOs [118]. *P53* KO work has not shown the ability to form pNENs alone, although they do create more aggressive pNECs in combination with Rb1 heterozygous KOs [119]. Finally, work to study heterozygous KOs of *Cul9*, a ubiquitin ligase of *p53*, demonstrated a case of pNEN formation in a cohort of mice at 21 months [120]. While heterozygous KO models of pNEN are a useful tool, the long development time coupled with expression of other tumor types makes them an inconsistent study model [116]. 

### 3.5. Conventional Germline Homozygous Knockouts

In contrast to heterozygous KOs, homozygous KOs are achieved through the complete targeted deletion of a functional gene. Targeted deletions are created through the breeding of heterozygous KOs to create the genotype. The genes must not be essential for embryogenesis or normal animal function, as these offspring would be non-viable. A common target in pNENs is hormone production related to the function of the pancreas (Table 6). As witnessed in RIP-Tag mice, altered insulin production causes rapid mortality, thus, homozygous inactivating models avoid insulinomas. Alterations to the pro-hormone convertase 2 (*SPC2* or *PC2*) using an inactivating neomycin-resistant gene insertion result in normal births but cause alpha and delta cell hyperplasia at three months followed by tumor development at six months [123,124]. Other groups have investigated the glucagon receptor (Gcgr) as a potential homozygous target. Similar to the *SPC2*-null mice, mice are born normally, but within 2–3 months have alpha cell hyperplasia, with tumors present at 10–12 months [125,126]. Finally, elimination of peroxiredoxin (PrDX1), a protein involved in antioxidant enzymatic activity, gave rise to mice with severe hematological abnormalities with a very high rate of cancer, although pNENs were in the minority of the cancers observed [127]. Homozygous deletion models are useful in monitoring the role of altered hormone production in mice although are insufficient for several genotypic profiles.

### 3.6. Induced Activation Models

Models of induced activation are utilized as a selective way to analyze the effect of potential oncogenes on tumor development (Table 7). Methods of induced activation include fusion to an insulin promoter sequence or usage of a doxycycline-inducible system. They can often be combined with other GEMMs, including KOs or Cre-Lox, to further amplify their findings. Generation of a fused RIP with a constitutionally active AKT by way of an added N-terminal myristoylation was able to demonstrate increased beta cell hyperplasia, which also was able to prevent diabetes mellitus in mice [128]. This research group would continue their work on Akt, linking the poor outcomes in mice to lower PTEN expression, and crossed the RIP-MyrAkt1 mice with S6 kinase 1 deficient mice, showing a lower rate of beta cell hyperplasia. Another group took advantage of using an insulin promoter (pIns) to create a reversible c-Myc GEMM [129]. Activation of c-Myc alone was not enough to overcome cell mediated apoptosis, so the group additionally crossbred to mice with upregulated Bcl-xL, which led to rapid beta cell survival, angiogenesis, and proliferation within weeks of activation, which was then reversed. An interesting GEMM model of virally introduced TVA, a receptor for avian leukosis sarcoma virus group A under the elastase promoter in a similar method as RIP-Tag mice, was described in 2003. Using this model, introduction of c-Myc into elastase-tv-a transgenic lnk4a/Arf-null mice would result in the development of insulinoma in approximately one-third of mice, in addition to other tumor types [130]. Analysis of increased pancreatic thymidylate synthase (TS) by way of a cytomegalovirus promoter demonstrated a significant increase of insulin positive islet hyperplasia and a low rate of adenoma when compared to control mice [131]. Increased activation of CDK5 was shown in patient tissues and in mice with a dox-inducible p25-GFP system found tumor development within 6 months of activation and showed *MEN1* heterogeneity that did not affect the increased CDK5 expression [132]. Most recently, one group combined the induced increased expression of TS with Cre-Lox-targeted *MEN1* expression to show how the two would create shorter survival in mice, in particular for homozygous KO vs. heterozygous KO [133]. The mice experienced faster pNEN progression, decreased expression of CDK-inhibitor tumor suppressors causing more rapid entry into cell cycle, increased chromosomal abnormalities, and increased somatic mutations. Overall, induced activation in GEMMs is a powerful tool for analysis of potential oncogenic phenotypes and can be combined with several other systems to create unique study models.

### 3.7. Homozygous Knock-In

Homozygous knock-in models are advantageous in the study of pNENs, where mutations have caused altered function (Table 8). The knock-in can be created by inducing a genomic alteration in single cell embryos, often point mutations at loci of interest, which are then incorporated into developing mouse embryos and crossbred to maintain mouse lines. However, as pNENs have a low mutational burden, there is less interest in creating knock-in models as compared to other GEMMs. An effort to create point mutations at *CDK* (R24C) demonstrated that cells became immortal, and a double mutation resulted in a wide range of tumors, including several pNEN subtypes in mice [135]. Another group created a GFP knock-in at the glucagon (*Gcg*) locus, creating an easily visualized model of *Gcg*-expressing cells in mice [136]. The double-mutant mice would be more susceptible to alpha cell hyperplasia, which was linked to increased Arx expression. Overall, work remains promising, but limited, in homozygous knock-in models.

While models of heterozygous or homozygous KOs have created numerous discoveries, the limitations of modeling germline mutations essential for organism development can prevent the analysis of certain genes, including homozygous inactivation of *MEN1*. Importantly, a system of controlled expression would allow for both a negative control and experimental model in a single animal, ensuring the change in expression is responsible for any alterations in the model organisms. The Cre-LoxP system was developed from the bacteriophage P1 and uses the Cre recombinase enzyme to create a recombination of a target sequence present between two LoxP sequences inserted into the genome, deleting the sequence [137]. A “guide” gene for incorporation of the target sequence is important and should be a target specific to pancreas endocrine lineage cells, such as glucagon, *Prdx1*, or insulin promoters. The target sequence can be either important in the repression or activation of genes. Importantly, this system can allow for the controlled activation by several methods, including tamoxifen induced (CreERT) or doxycycline (Dox). In generating Cre-LoxP mice, two parental strains need to be generated: one with the Cre-driver strain in which Cre recombinase will target the cell or tissue of interest and another with the loxP flanked (floxed) DNA. The two are then bred to create the conditionally transformed mice. Since the inception of the technology, it has been used in pancreatic cell origin, diabetes, and pNEN research [138].

Overwhelmingly, pNEN work with the Cre-LoxP system has involved alterations to *MEN1* (Table 9). The first published work of Cre-LoxP in pNENs involved the conditional deletion of *MEN1*, an important target of pNEN research, in 2001 [117]. Breeding of embryonically created heterozygous KOs resulted in no viable homozygous offspring, while heterozygotes developed normally but began forming tumors at 9 months. A similar study was published in 2003, where embryonically heterozygous *MEN1* mice were shown to develop insulinomas and glucagonomas in addition to many other *MEN1* deficient associated tumor types [139]. The first pancreas-specific model of Cre-LoxP deletion for MEN1 homozygous deletion in 2004 demonstrated complete penetrance of hyperplasia in beta cells, and crosses with RIP-Cre^+^ mice would also produce anterior pituitary gland adenomas [140]. Cre-LoxP deletions of a copy of *MEN1* and *Rb1* were generated independently and crossed to create double-heterozygous mice for *MEN1* and *Rb1*, which generated higher tumor burden of multiple tumor types and islet hyperplasia but did not decrease survival significantly than either *MEN1* or *Rb1* only heterozygotes [118]. Cre targeting of *MEN1* in pancreatic progenitor cells using a Pdx1-Cre recombinase demonstrated endocrine only pancreatic insulinoma development at 10–12 months, resulting from increased VEGF expression, which could be reduced by anti-VEGF therapy [141]. An alpha-cell-specific MEN1 homozygous knockout was described using the glucagon promoter and showed increased expression of glucagonomas during 2–3 months of age [142]. Curiously, insulinomas also arose in late development, which was determined to be from the dysregulation of pancreas progenitor cell development into beta cells. Simultaneously, another group would confirm these findings in a Cre-LoxP heterozygous and homozygous alpha-cell deletion model of *MEN1* [143]. A combined Cre-LoxP model targeting *MEN1* and β-Catenin would cause a decrease in tumor formation and aggressiveness when compared to *MEN1*-alone deletion, demonstrating a role for β-Catenin in tumorigenesis for these mice [144]. Another MEN1 deletion model was described using a beta-cell-specific, tamoxifen-induced RIP2 promoter [145]. Mice developed beta cell hyperplasia within two months and showed complete lack of menin. A cross of floxed *PTEN* and *MEN1* using RIP-cre was performed and determined a synergistic effect of the two KOs in developing G1/G2 well differentiated pNENs [146]. However, this group also used mouse insulin promoter (MIP)-cre mice, which do not form pituitary NETS like the RIP-cre mice and showed pancreas-only tumor development at a similar rate as the RIP-cre mice. Most recently, a Cre-LoxP system guided by GFAP cellular expression used to target *MEN1* with a tdTomato reporter was generated [147]. The elimination of *MEN1* alone was sufficient to form pNENs in addition to other expected tumor types, including pituitary adenomas; however, the researchers would further analyze the mice by also blocking somatostatin and hedgehog pathway mediator KIF3A, demonstrating the blockage of the two could reverse islet hyperplasia in *MEN1*^−/− ^pancreas. A model of *MEN1* deletion targeted to SOX10 expressing cells would also create pNEN development in this study. Efforts have demonstrated extensive work for multiple cell types in MEN1 deletions, providing valuable insights into tumor cell origin and fate.

In addition to MEN1, Cre-LoxP has been used to target other genes for elimination (Table 10). One group developed a *VHL* pNEN model through homozygous Cre-LoxP deletion of *VHL* using a glucagon promoter, showing islet dysmorphia and hyperplasia but mostly demonstrating exocrine pancreas tumors [149]. A Cre-LoxP model targeting renin-based p53 and Rb1 demonstrated a high penetrance of metastatic glucagonomas along with subcutaneous tumors [150]. The deletion of fibronectin using a tamoxifen activator was performed in a study of RIP-Tag mice to determine the effect on tumor growth and angiogenesis [151]. While angiogenesis was delayed in these mice, islet cell growth or mass was not affected by the deletion. A comprehensive study involving Cre-LoxP deletion of *Ink4a/Arf*, *p53*, or a combination of the two in RIP7 mice with the viral oncogene PyMT demonstrated an increased rate of beta cell pNENs when the two genes were deleted in the same mouse [152]. Usage of Cre-LoxP eliminated *Rb1* allowed for the development of WD pNENs within 18–20 months and the crossing of these mice with Tp53 mutation showed a significant increase in number of tumors and decrease in time of formation to 6 months, although the Tp53 mutation alone was not sufficient to form pNENs [153]. This was shown to be related to the decrease in PTEN, a regulator of the mTOR pathway, which could be targeted to reduce tumor size. Models using Cre-LoxP targeting have shown robust utility for tumor suppressors, and further work will provide insight into pNEN development and behavior.

### 3.8. Other Animal Genetic Models in PNEN Research

Outside of genetic mouse models, a variety of other species have been utilized in pNEN research (Table 11). Rats are one of the original models of induced pNENs and have been in use since the 1970s. The first published model was an strain of NEDH inbred albino rats which were given full body X-ray radiation [61]. The resulting tumor was then xenografted into a large line of other rats, resulting in a long-term stable line which has been used in the generation of other models [45,46]. Another example of induced tumorigenesis in a rat was performed using streptozotocin and nicotinamide, a former diabetes treatment [154]. Islet cell tumor development was widely variable but showed near complete penetrance over 24 months. The SV40 Tag was used in a pair of studies analyzing the development of neuroendocrine tumors on Sprague–Dawley rats [155,156]. Tumor development occurred at 3–5 months, although the a/b SV40-Tag rats had a higher rate of gastric neuroendocrine tumors, while the pPEPCK promoter-SV40-Tag demonstrated complete penetrance for pNENs. Using the pPEPCK promoter, TGF-Alpha was also targeted for increase, which demonstrated another islet cell carcinoma pNET model of complete penetrance with no other tumor anomalies [156]. Another animal model of interest is the zebrafish, which has numerous biological advantages, including translucent skin, similarity of pancreas cellular makeup, ease of genetic manipulation, and rapid, high-throughput capabilities [157,158]. While there are zebrafish models for common neuroendocrine tumor genomics, including *MEN1, VHL, DAXX*, and *ACTH*, only one model of pNEN tumor development has been published. A model of MYCN overexpression was developed to understand how tumor progression occurs in zebrafish [159]. Over the course of 6 months, 1.6% of the altered fish developed GFP^+^ islet cell tumors, including NF, insulinomas, and glucagonomas. Further work may continue these efforts for the other genetic models of zebrafish. Like zebrafish, frogs (X. tropicalis) have many of the same advantages, including a well-developed genetic modification toolbox [160]. A study analyzing the relationship of *Rb1, rbl1*, and *p53* in CRISPR-Cas9 KOs demonstrated that *p53* did not enhance tumor growth in the *Rb1/rbl1* KOs for pNEC SC tumors [161]. Finally, although there are no established individual models for pNENs, canine insulinomas mimic the sporadic, long-term development nature of humans and have similar mutations including *MEN1*, *ATRX,* and in SSTR2 expression [162]. Indeed, the tumors are present in multiple dog breeds and may lead to further understanding of pNEN development in humans [163]. Overall, while future work is needed, the ability to create high-throughput, easily manipulatable strains (zebrafish and frogs) and sporadically occurring, long development tumors (dogs) may lead to significant breakthroughs soon.

## 4. 3D Models of pNENs 

**Figure 4:**
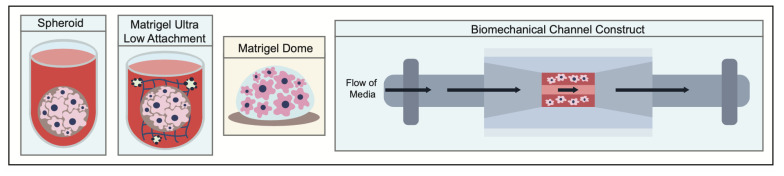
Side by side comparison. From left to right: spheroid, ultra-low attachment assisted organoid, ECM-supported organoid, and bioreactor-based model.

Three-dimensional tumor models are the intermediate step in terms of complexity and utility when compared to both traditional 2D cell line and animal models. They represent a middle ground in terms of accuracy, function, cost, and throughput. Three-dimensional models of tumors can be broadly summarized into three categories: spheroids, extracellular-matrix (ECM)-supported, or bioengineered hybrid models (Figure 4). Herein, we will cover human 3D models of pNENs (Table 12). 

### 4.1. Spheroids

Spheroids consist of adhered cells forming a collective, spherical unit. They are typically formed through low-gravity environments such as the hanging-drop technique or with special plates to mimic low gravity [180]. Spheroids have improved cell–cell interactions and have been shown to better mimic therapy response compared to 2D cultures [181]. Due to the fewer cells needed to form, they are capable of rapid scaling for therapeutic testing and simple mechanistic analysis [182]. However, they have a size limit due to the gradient constraints of nutrient availability and little native ECM content, which caps their complexity. They are also limited by the types of cells used, as not all cell populations are capable of spheroid formation; indeed, the formation ability of spheroids is used as a method to test stemness in primary tumor tissues [183].

The overarching theme of spheroid usage in pNEN research is in drug screening applications. The first 3D modeling of human pNENs was performed in 2012 by Wong et al., who described spheroid formation using BON-1 and QGP-1 cells in agarose-coated plates for usage in a single-drug therapeutic screen, Trichostatin A (TSA), and demonstrated a smooth spheroid formation for BON-1 but not for QGP-1 [164]. The same group would also demonstrate utilizing dual-treatment Rapamycin and CPI203, a BET inhibitor, to reduce MYC activation and decrease cell growth in 2D, spheroid, and mouse xenograft models [165]. The effect of radiation on BON-1 agarose-derived spheroids was performed in 2018, determining they were an effective model for the measurement of apoptosis and cell cycle arrest after irradiation [166]. Combination of an HSP90 inhibitor and 177Lu-DOTATE was performed in BON-1 spheroids, elucidating a synergistic effect by increasing DNA damage and increased apoptotic signaling in relation to SSTR2 signaling [168]. A comparison of three spheroid formation methods, the hanging drop method, 24-well cell repellent plates, and 96-well ultra-low attachment (ULA) plates, was performed on BON-1 cells to determine the advantages of each [167]. Through several analyses, including spheroid formation, shape, size, and sunitinib treatment, the group suggested the use of ULA plates was the most efficient and easiest method of culture for BON-1 spheroids. Cell lines BON-1, a mouse-derived insulinoma cell population, and the pancreatic stellate cell line HPSC2.2 were utilized to assess the efficacy of deacetylase therapy to increase autophagic cell death. The group demonstrated a decrease in AMPK activation and an increase in autophagosome accumulation in both tumor cell lines, which was linked to patient outcomes [169]. Two-dimensional and 3D cultures of BON-1 and QGP-1 cells were tested using telotristat, a molecule which alters serotonin production was performed in 2020 [170]. The authors noted the compound was able to decrease serotonin production without reducing cell viability and could be combined with SSTR2 analogs to further reduce serotonin production, which could help in the treatment of functional pNETs. Work to analyze the role of *MEN1* in the expression of apoptosis markers was performed in BON-1 and QGP-1 spheroids, demonstrating a negative link between *MEN1* expression and TP53 and CDKN1A, which could be remedied with staurosporine [171] Finally, work to analyze dual PI3K and CDK4/6 inhibition was performed on three pNEN cell-line spheroids (BON-1, INS-1E, and NT-3), along with four patient specimens. Showing that the two agents, buparlisib and ribociclib, worked in synergy to reduce downstream proteins AKT and Rb in all models tested. Overall, spheroids have served to model cell lines in pNENs, with the opportunity for primary cell incorporation on the horizon.

### 4.2. ECM-Supported Constructs

ECM-supported constructs, including organoids and tumoroids (PTOs), can form larger and more complex cultures using ECM, which provide an increase in nutritional support while better mimicking cell–cell interactions [184]. They function as rudimentary organs, often maintaining similar functions as the tissues from which they are derived. The ECM materials utilized often include basement membrane matrix, such as Matrigel, or combining ECM components including collagen, hyaluronic acid, or others [185]. Importantly, they provide sites for cellular interactions and may be supplemented with growth factors. These constructs allow for incorporation of other cell types, including stromal and immune cells, to provide more accurate representation of cellular populations in tissues [186]. Additionally, due to their utility in modeling cells from patient tissues, they have found a role in accurately modeling tumor cell populations and tumor microenvironment interactions in a range of tumors, including pancreatic, neuroendocrine, and other rare tumors [187,188,189,190,191,192]. They are also capable of accurately representing patient treatment response, with multiple studies correlating results between organoids and patients and predicting clinical trial outcomes [193,194,195]. While they provide complexity when compared to 2D culture, they do not provide the multi-organ interactions witnessed in animal models. Additionally, Matrigel is xenobiologic and contains components which make expansion into the clinical setting a challenge [196]. Finally, despite the historically increased chance of engraftment when compared to PDX models, PTO success has remained limited, often due to slow growth of pNENs, especially when creating a pNEN model of G1 or G2. 

There are currently four published works describing PTO work with two preprints in press. Kawasaki et al. described the first study using PTOs to represent pNENs in a larger study of neuroendocrine tumors [82]. They were able to model one G3 pNET and two pNECs out of a total of eight tissues (37.5%) for several passages, with each procured through biopsies. Of the eight attempted, four of the failures were G1 or G2, with all successful attempts having a Ki67 index of >35%. The group noted mutations to *TP53, RB1, DAXX1*, and *KRAS*, with large chromosomal alterations to the pNET PTO group. Finally, the group analyzed growth factor dependency based on PTO mutational profiles and used CRISPR/Cas9 technology to solidify these findings. The second published study modeled pNENs as tumoroids using cryopreserved specimens including insulinomas and NF pNENs, demonstrating the potential for a multicenter approach [173]. The group described tumor cell isolation success in 8/11 (72.7%) of specimens, with a further 6/7 (85.7%) able to provide two weeks of viability with successful drug screening data. A low ki67 index was noted for some matched tumoroids and tissues, demonstrating the potential to culture lower grade tumors using ultra-low attachment plates to assist Matrigel organoid formation. Finally, the group utilized clinical compounds, including sunitinib, everolimus, and temozolomide, to determine therapeutic sensitivity, noting variable responses based on tumoroid testing. Shi et al. would establish a large bank of Matrigel PTOs from both exocrine tumors and pNENs to analyze the link between chromatin accessibility and therapeutic response in pancreatic cancers [174]. pNEN subtypes involved in this study included G1, G2, and pNECs. The analysis performed was able to create distinct groups of pancreatic tumors through transcription factors, predict both chromatin accessibility peaks and potential cancer driver mutations, and even create a therapeutic correlative model based on these characteristics. Lastly, Hogenson et al. utilized pNET tissues for organoid establishment as a part of a larger study in establishing gastrointestinal PTOs to compare two widely used media compositions: WNT based culture medium and PaTOM [175]. The authors claimed a success rate of 53.8% (7/13) for PTO formation, although three lines were unable to reach passage three due to slow growth or fibroblast contamination. Establishment success was greater for WNT based media composition (3/3 PTOs attempted, 100%) when compared to PaTOM (2/11, 18.2%). Immunohistochemical characterization was performed on one of the PTO lines, although they were not further used for treatment sensitivity screening or for patient correlative studies presented later for PDAC patients.

Two preprints further demonstrating robust value to PTO research are currently in press. Dayton et al. has described the establishment of a variety of neuroendocrine PTOs, which include one large-cell pNEC [176]. They noted the inverse link between passage time and tumor grade and were able to determine IHC, gene, and transcriptomic expression similarities between the PTOs and the parent tissue, including SSTR2. The researchers noted the PTOs contained a clonal *TP53* mutation which was derived from the parental tumor but were also able to determine the pNEC PTOs showed large intra-tumor diversity compared to other neuroendocrine tumor PTOs in this study. Finally, drug screening applications were demonstrated using paclitaxel, everolimus, and navitoclax, demonstrating the suitability of these PTOs for this application. The second preprint demonstrated the continued work of April-Monn et al. on tumoroids [177]. The group would create three new PTO sets from pNEN tissues, although a rigorous preselection process was performed beforehand. The tumoroids tested represented several mutation profiles, including *DAXX*, *ATRX*, and *Rb1*, with all containing >75% Ki67 expression. The study also confirmed the maintenance of gene expression pathways, including *Myc, p53*, and *EGF/VEGF,* between tumoroids and PTOs. Interestingly, QGP-1 and NT-3 spheroids were also created in this study and compared to the primary tissues, demonstrating differences in gene expression and showing the tumoroids were more accurate. Tumoroids were then screened with cisplatin or temozolomide, illustrating patient specific responses which could match the clinical courses of the patients who were treated similarly. Further analysis of treated tumoroids would suggest IFNB1 and KDM5A as targets for combinational therapy, which the authors confirmed with inhibitors for each target as well as with cisplatin in tumoroids and in a QGP1 xenograft in zebrafish. Current advances in 3D ECM supported constructs have demonstrated notable promise in modeling cancers throughout the body, and new research continues to advance our understanding of pNENs.

### 4.3. Bioengineered Hybrid Models 

Bioengineered hybrid 3D models contain both biological and artificial components to functionally mimic the tumor microenvironment. These components may include microfluidics to represent circulation or channels to create tumor “invasive” potential [197]. They may also incorporate other technologies to allow for standardization, such as bioreactors, bioprinting, molding, or laser etching [198]. There has been considerable interest in developing tissue-mimetic bioengineered substrates, and organs, such as the heart, bone, liver, and pancreas, have had considerable efforts to model. Due to these technologies, the complexity of these models can be increased substantially, with even the capability of linking multiple representative organ systems [181]. However, as many of these technologies are used in proprietary ways, throughput remains low, with significant standardization needed to scale them to reproducible levels.

One group has described using bioreactor technology to keep patient tissue slices of neuroendocrine tumors, BON-1 cells, and BON-1 PDX tumors alive for over a month. Herring et al. created a joint hydrogel and polydimethylsiloxane (PDMS) bioreactor attached to inflow and outflow channels, which allowed for flow of cell culture media to mimic vascularization. In their first study, they confirmed viability for each tissue group and demonstrated the ability to track therapeutic response in pretreated tissues [178]. The researchers would follow up by expanding the parameters measured to include imaging and flow cytometry and increased the number of therapeutics tested, including radiation and thailandepsin-A, a histone deacetylase [179]. The group was able to validate their system and confirm growth of embedded pNET tissue for over three weeks and even passaged their system to seed another bioreactor, demonstrating the capability of expandable growth. While there is only one type of model in the current literature, there is opportunity to further expand and integrate other technologies into pNET modeling.

## 5. Discussion

pNENs have experienced profound interest from the clinical community due to the rise of cases and lack of mechanistic understanding. Combined, the outcomes for these patients remain poor, with subtype heterogeneity confounding treatment prognosis and preventing clinical trial accrual. To improve these issues, preclinical models must create an accurate and reproducible system to analyze the biology behind these rare tumors. Researchers must balance model complexity with high-throughput capability. Advancements in technology have allowed for focused, controlled study systems which have improved our understanding of individual subtypes; nevertheless, there is ground to be made to improve the outlook of pNENs. 

Considerations for study model choice must include both advantages and pitfalls of each option. Cell line models of pNENs carry the advantages of high throughput and low cost. Experience with lines has carried through decades of pNEN research and they have been involved in the development of many clinical agents. However, there are significant shortcomings in the modeling of pNETs, as most derived cell lines undergo significant oncogenic transformation resulting in a highly proliferate, mutation-heavy profile seen in pNECs. Cells grown in 2D culture are not representative of cells grown in the body as they respond to different spatial stimuli. Additionally, there has been recent debate about the evolution of established lines, with some lines falling out of favor as more advanced analyses finds tumor characteristics related to non-pNEN diseases or the discovery of shifted mutational profiles in established cell lines [57,67]. Animal models have a robust history in pNEN modeling, and a variety of generation techniques are currently available for model development. Over time, these techniques have increasingly become more focused and precise in mice, leading to the accurate modeling of both spontaneous tumors and hereditary linked mutations, including *MEN1*. As whole organisms, the interactions between the pNEN and the entire body, including nearby and distal organ systems along with non-cellular ECM components, can be observed. These relationships also allow for more accurate therapeutic testing, as the effects of the therapy, from dosing to on-target tumor and off-target healthy tissues can be measured. Patient-derived xenograft establishment also provides another method of long-term propagation of primary tumor cells, allowing for continued study. Current interest in non-mouse models, including zebrafish and frogs, demonstrates opportunities for models of advanced genetic manipulation and have shown this promise in other tumor types. However, being non-human models, there can never be complete translational comparison as animals often contain different versions of the genes present in humans. Many methods of genetically manipulated animal models suffer from either slow, unpredictable tumor development, off-target effects, or low success rates. Additionally, high institutional upkeep and lower throughput prevent large scale implementation of many models as a universal standard. 

Three dimensional cellular models are the newest arrival to the pNEN research benchtop. These models allow for spatially accurate systems of cellular interactions, which has been shown to improve tumor signaling along with cell phenotype and genomics [199]. Recently, the utilization of patient derived organoid culture has allowed for successful growth of ex vivo cultures of pNEN cells from a variety of grades and subtypes with a high degree of fidelity to the original patient. Tissues from patients can be cryopreserved and sent to different institutions for later fabrication, and cultures can be frozen and shared across in the same manner. The inclusion of bioengineered components including artificial blood flow and distal healthy sites may allow for improved tumor migration and invasion testing. Current weaknesses for 3D cellular models are the limited accrual for rare cancer specimens which plagues many centers, coupled with the relatively non-standardized methods of culture between centers; this has prevented comparison of results. Additionally, while they are more accurate than 2D cultures, they remain limited by the site of acquisition, as new research shows the importance of tumor heterogeneity in specimens taken from intratumor locations [200]. In designing studies investigating complex molecular interactions, it would be prudent to involve multiple study models to utilize the advantages of all and overcome shortcomings of each.

## 6. Future Directions

Further advances in technology have shown promise in continuing to better model pNENs. Most importantly, recent advances in our understanding of genetics, proteomics, and epigenetics have allowed for a significant increase in model fidelity and number. Several recent cohorts of genetic analyses in pNEN patient populations have increased our knowledge level, identifying new genotypes and potential targets [12,14,21,201]. These approaches, combined with a new precision medicine oncology approach observed in many other cancers, can create a more targeted and optimized treatment plan for patients. Advances in gene editing, including CRISPR/Cas9, have been utilized in both cell and in frog models of pNENs, creating new molecular model subtypes of previously existing models [36,161,202]. Coupled with more advanced maintenance and establishment techniques, new cell lines and 3D models have shown to be more accurate representations of pNENs than earlier established methods [57,177]. Three-dimensional models are especially promising, creating an accurate tumor model while combining the high-throughput capabilities of cell lines. Industrialization efforts shown in other rare cancers such as bioprinting can increase this capability and allows for the development of more complex 3D models [203]. Finally, institutional collaboration, including biobank establishment and resource sharing, can increase accruement and distribution of these valuable tumor samples, increasing the significance of research findings [204]. The creation of these programs, potentially with the support of a large institutional program grant, would lead to consequential advancements in pNEN research, including genetic, transcriptomic, and therapeutic understanding which can improve the outcomes of pNEN patients.

## 7. Conclusions

pNENs are a complex family of tumors due to their slow growth and rarity. Despite this, pNEN model development has progressed substantially, with increasingly complex models being developed. With new technological advances, future models will continue this trend. 

## Figures and Tables

**Table 1:** List of Published Cell Lines. NA—not available. Met—metastasis.

Cell Name	Year	Species	Tumor Subtype	Primary/ Metastasis	Notable Features or Mutations	References
βTC	1988	Mouse	Insulinoma	Primary	SV40 T-Antigen in RIP-Tag Mice	[37]
MIN6	1990	Mouse	Insulinoma	Primary	SV40 T-Antigen in transgenic mice	[38]
Alpha TC1	1990	Mouse	Glucagonoma	Primary (Alpha Cell)	PR proglucagon promoter-driven SV40 T antigen from C57BL/6 x DBA/2 mouse	[39]
BTC	1990	Mouse	Insulinoma	Primary	Polyoma small T antigen and SV40 from C57BL/6J double transgenic RIP1Tag2/ Rip2pyST1	[40,41]
NIT-1	1991	Mouse	β-Cell Adenoma	Primary	NOD/Lt mice insulin-promoter/SV40 T-antigen	[42]
TGP61	1994	Mouse	Insulinoma	Primary	Tg(Ela-1-SV40E)Bri18 transgenic mice with SV40 T antigen	[43]
Mu Islet (E6/E7)	2009	Mouse	NA	Primary (Alpha Cells)	C57BLKS/J Mouse Islet cells transduced by HPV E6 and E7 antigens	[44]
RIN	1980	Rat	Insulinoma	Primary	X-ray induced Inbred NEDH rat strain	[45]
INS1	1992	Rat	Insulinoma	Primary	X-ray induced Inbred NEDH rat strain	[46]
HIT	1981	Syrian Hamster	Primary Islet Culture	Primary	Islets transformed from SV40	[47]
canINS	2017	Dog	Insulinoma	Primary	NA	[48]
QGP-1	1980	Human	Somatostatinoma Delta Cells	Primary	*ATRX, KRAS, TP53, APC*	[49]
CM	1987	Human	Insulinoma	Met (Ascites)	Severe Chromosomal Aberrations	[50]
BON-1	1991	Human	NF pNEC	Met (LN)	*ATRX, TSC2, NRAS, TP53, BRCA2, APC, CDK2A/B*	[51]
HuNET	2001	Human	VIP	Primary	NA	[52]
A99	2011	Human	SCC	Met (Liver)	*p53*, *KRAS*	[53]
APL1	2016	Human	NF, G1	Met (Liver)	CD47+, HGF/MET	[54]
NT-3	2018	Human	WD Insulinoma	Met (LN)	SSTR+, *MEN1*, VEGF+	[55]
SPNE1	2022	Human	pNET	Primary	CD44+, SSTR+	[56]
NT-18P	2022	Human	G3	Primary	*DAXX, MEN1, p53, MSH6*	[57]
NT-18LM	2022	Human	G3	Met (Liver)	*DAXX, MEN1, MSH6*	[57]
NT-36	2022	Human	G3	Primary (recurrence)	*DAXX, RAD50, MEN1, MSH6*	[57]
NT-32	2022	Human	LC pNEC	Primary	*BRAF, RB1, p53*	[57]

**Table 2:** Patient-Derived Xenograft Research in pNENs. Subcu—subcutaneous, inj—injection. * One passage.

Author	Year	Host Species	Strain	Tumor Type	Xenograft Source	PDX Method/Site	Success Rate	References
Yang	2016	Mouse	NOD-SCID	NET	Tumor	Subcu flank Tissue	3/58 (5.2%) *	[80]
Chamberlain	2018	Mouse	Athymic Nude	Insulinoma (liver met)	Tumor	Subcu Tissue	1/1 (100%)	[81]
Kawasaki	2020	Mouse	NOG	NETG3, NEC (LC)	Organoid	Renal/spleen	2/2 (100%)	[82]
Pham	2020	Mouse	NOD SCID	pNET	Tumor	Subcu, Orthotopic	1/5 (20%)	[83]
Tran	2022	Mouse	NSG	NETG2	Tumor	Subcu Cells	0/3 (0%)	[84]
Gaudenzi	2017	Zebrafish	Tg(fli1a:EGFP) ^y1^	NETG1 (LM)	Tumor	Embryo injection (cells)	1/1 (100%)	[85]

**Table 3:** RIP-Tag mouse models. Many >5 other tumor types. NA = not available.

Model Name	Year	pNEN Subtype	Target Gene	Strain	Model Type	Tumor Development	% of Mice w pNENs	Other Tumors	References
RIP-Tag	1985	Beta Cell Tumors, pNEC	SV40 Tag (RIP)	B6D2Fl (C57BL/6J × DBA/21	Transgenic	10–20 weeks	100%	None	[87,102,103]
RIP1Tag2/RIP2PyST1	1990	Beta Cell Tumors	SV40 Tag and polyoma small T-antigen (PyST)	RIP-Tag Mice	Transgenic	6–14 weeks	100%	Colon	[40,41]
RIP-Tag5	1996	Beta Cell Tumor	*SV40 Tag* (*RIP*)	C3Heb/FeJ (CH3)	Transgenic	17–22 Weeks	100%	None	[101]
*Rip1Tag2xRip1E-Cad, Rip1Tag2xRip1dnE-cad*	1998	Beta Cell Tumors	SV40 Tag (E-Cad)	B6D2Fl (C57BL/6J × DBA/21	Transgenic	10–16 Weeks	7.8%, 50.6%	None	[96]
RipVEGF-C × Rip1Tag2	2001	Insulinomas	SV40 Tag (RIP) and VEGF-C (RIP)	B6D2Fl (C57BL/6J × DBA/21	Transgenic	10 Weeks	100%	None	[89]
Rip1Tag2/Rip1VEGF-A	2002	pNETs	SV40 Tag (RIP) and VEGF-A (RIP)	B6D2Fl (C57BL/6J × DBA/21	Transgenic	10 weeks	100%	None	[90]
RIP7-Igf-1R, RIP1-Tag2	2002	Invasive Carcinoma, Beta Cell Hyperlplasia	SV40 Tag (RIP) and IGF1R (RIP)	B6D2Fl (C57BL/6J × DBA/21	Transgenic	5–15 weeks	100%	None	[92]
*RIP-Tag2,Rag^−/−^*	2005	Beta Cell Hyperplasia	Rag1	C57-B16-J	Transgenic, Homozygous KO	13.5 Weeks	100%	None	[95]
Rip1Tag2;Rip1VEGF-D	2007	Beta Cell Tumors	SV40 Tag (RIP) and VEGF-D (RIP)	C57BL/6	Transgenic	12–14 Weeks	100%	None	[91]
RipTag-IRES-Luciferase (RTL) 1	2010	Insulinoma	SV40 Tag (RIP-IRES)	C57Bl/6	Transgenic	7–10 weeks	100%	None	[88]
RT2/TNC, RT2/TNCKO	2013	Insulinoma	TNC	C57Bl6, C57Bl6 × 129/Sv-C57Bl6	Transgenic	8–12 weeks	NA	None	[93]
*Hpa*-Tg RT2, *Hpse^−/−^* RT2	2013	Islet Cell Carcinoma	Heparanase	C57BL/6	Transgenic, Homozygous KO	13.5 weeks	100%	None	[94]
RIP-Tag2	2019	NF pNETs	SV40 Tag (RIP), Insm1 low	RT2 AB6F1	Transgenic	17 weeks	100%	None	[98]
*RIP-TAG*	2020	pNET	RIP-Tag	RT2 B6A(F1)	Transgenic	8–12 Weeks	100%	siNET	[99]
RIP-TAG2, pl-PDGFB KO	2022	Islet Cell Carcinoma	RIP-Tag2, PDGFB	C57BL/6	Transgenic, Selective KO	8–14 Weeks	100%	None	[97]

**Table 4:** SV40-Tag sequences other than RIP. Many >5 other tumor types. NA = not available.

Model Name	Year	pNEN Subtype	Target Gene	Strain	Model Type	Tumor Development	% of Mice w pNENs	Other Tumors	References
VT-C (AVP-Tag)	1987	Islet Dysplasia	SV40-Tag (Vasopressin)	C57B1/KJ X SJL F1	Transgenic	90–140 Days Hyp	7/8	Pituitary	[111]
ESLV Tg (Ela-1,SV40E)Bri18	1987	D-Cell Hyperplasia, Insulinomas	SV40-Tag (Elastase)	C57/SJL F2	Transgenic	8 weeks Hyp 20 Weeks Tumors	100%	Pancreatic Exocrine Tumor	[104,105]
Glu2-Tag	1988	Alpha Cell Hyperplasia	SV40-Tag (*Glu2*)	C57BL/6J × DBA/2J	Transgenic	5 months Hyp, 9–12-months tumors	100%	None	[109]
SV-202	1989	Islet Cell Adenoma	SV40-Tag (MT)	C57BL/6JXSJL F1	Transgenic	15 Weeks tumors, 20 Weeks (Death)	100%	Liver	[112,113]
MSV125	1990	Insulinoma	MSV-SV40	NA	Transgenic	2–12 Months	100%	Brain, Eye, Kidney, Sarcoma	[106]
L-PK/Tag	1992	Islet Cell Carcinoma GLUTag-Ytg	SV40 Tag (L-Type Pyruvate-Kinase)	(C57BL/6 × DBA)F1	Transgenic	NA	80%	Liver	[107]
GLUTag-Ytg	1992	Islet Cell Carcinoma	SV40 Tag (*RG*)	CD1	Transgenic	11–12 weeks	100%	Colon	[110,114]
GP1.5 Tag, GP10.5 Tag	1993	Pancreatic Islet Cell Tumors	SV40 Tag (*Gastrin*)	CD1	Transgenic	80–100 Days (Death)	100%	Hepatobiliary Tract	[108]
Secretin-Tag	1995	Insulinoma	SV40 Tag (*Secretin*)	B_6_D_2_F_1_× B_6_D_2_F_1_ embryos (CD6)	Transgenic	12 Weeks	>80%	siNET, Colon	[115]

**Table 5:** Global heterogeneous knockouts in pNEN GEMMs. Many >5 other tumor types.

Model Name	Year	pNEN Subtype	Gene	Strain	Model Type	Tumor Development	% of Mice w pNENs	Other Tumors	References
Rb1^+/^p53^+/−^	1994	Islet Cell Tumors (pNEC)	*Rb1, p53*	(C57BLx CBA) × C57BL/6	Global Heterozygous KO	9 months, 3–6 months	14%, 23%	Many	[119,121]
MEN1^+/^^−^Rb1^+/^^−^	2007	Islet Cell Tumors	*MEN1, Rb1*	C57BL/6j:129 × FVB/N:129	Global Heterozygous KO	402 days	55%	Many	[118]
Men1^+/1^	2009	Insulinoma, Glucagonoma, NF	*MEN1*	C57BL/6	Global Heterozygous KO	9–12 months	60%	Many	[122]
Cul9^+/−^	2011	Insulinoma	*CUL9*	BL/6	Global Heterozygous KO	21 months	1/23 (4.3%)	Many	[120]

**Table 6:** Homozygous Global Knockouts of pNEN GEMMs. Many >5 other tumor types.

Model Name	Year	pNEN Subtype	Target Gene	Strain	Model Type	Tumor Development	% of Mice w pNENs	Other Tumors	References
Rb1^+/−^p53^−/−^	1994	Islet Cell Tumors (pNEC)	*Rb1, p53*	(C57BLx CBA) × C57BL/6	Global Heterozygous KO	3–6 months	14%, 23%	Many	[119,121]
SPC^−/−^	1997	Alpha and Delta Cell Hyperplasia	*SPC*	C57BL/6J	Homozygous KO	3 Months	100%	None	[123]
Gcgr^−/−^	2003	Glucagonoma	Gcgr	C57BL/6J	Homozygous KO	8 weeks (hyperplasia)	100%	None	[125]
Prdx^−/−^	2003	Pancreatic Islet Cell Adenoma	*PrDX1*	B6	Homozygous KO	9 months	9%	Many	[127]
Gcgr^−/−^	2011	Glucagonoma, NF	Gcgr2	C57BL/6 × DBA1/lacJ	Homozygous KO	5–7 months, 10–12 months	100%	None	[126]
PC2^−/−^	2014	Glucagonoma	*PCKS2*	C57Bl6	Homozygous KO	3 months (hyper) 6–8 months	100%	None	[124]

**Table 7:** pNEN GEMMs of Induced Activation. IA = inactivation. KO = knockout. Many >5 other tumor types. NA = not available.

Name	Year	pNEN Subtype	Target Gene	Strain	Model Type	Tumor Development	% of Mice w pNENs	Other Tumors	References
RIP-MyrAkt1	2001	NET	*MyrAKT (pS473)* (RIP)	B6SJLF1/J	Transgenic, IA	8–12 weeks (hyperplasia)	100%	NA	[128]
*pIns-c-MycER^TAM^/BCL-XL*	2002	Islet Cell Carcinoma	*MYC, BCL-xl* (*RIP*)	(CBA × C57BL/6)F1	Transgenic, IA	2 Weeks (post activation)	100%	None	[129]
Elastase-tv-a;RCAS-c-myc;p16^−/−^p19^-/-^	2003	Insulinoma	*c-Myc*	FVB (lnk4a/Arf null)	Transgenic, IA	7 months	4/14	Sarcoma, Lymphoma	[130]
TS-T1	2007	Islet Cell Tumors	Thymidylate Synthase	FVB	Transgenic, IA	9–24 months	23% hyper, 6% adenoma	None	[131]
RIP-MyrAkt1 (SK61^−/−^)	2008	Insulinoma	Akt1 and (S6K1)	B6SJLF1/J × (C57Bl/6xDBA/2)	Transgenic, IA	1 year (death)	19/23	Lung, Pancreatic Carcinoma	[134]
INS-p25OE	2021	Beta Cell (WD)	*CDK5R1*	Ins2-rtTA × tetOp-p25GFP	Transgenic, IA	10–15 weeks	100%	None	[132]
*hTS/Men1^–/–^*	2022	Islet Cell Carcinoma	Thymidylate Synthase, *MEN1*	FVB × (C57BL/6J × NIH Black Swiss females or 129/SvEvTacFBR) × (129/Ola × 129/Sv)	Transgenic, IA, Inducible homozygous KO	10 months	100%	Pituitary	[133]
*hTS/MEN1^+/−^*	2022	Islet Cell Carcinomas	Thymidylate Synthase, *MEN1*	FVB × (C57BL/6J × NIH Black Swiss females or 129/SvEvTacFBR) × (129/Ola × 129/Sv)	Transgenic, IA, Inducible heterozygous KO	22 months	100%	Pituitary	[133]

**Table 8:** Homozygous Knock-Ins of pNEN GEMMs. KI = knock-in. Many >5 other tumor types.

Name	Year	pNEN Subtype	Target Gene	Strain	Model Type	Tumor Development	% of Mice w pNENs	Other Tumors	References
*Cdk4* ^R24C/R24C^	2001	Beta Cell Tumor, PP, Glucagonoma	*Cdk4* *(R24C)*	mixed 129/Sv CD-1	Homozygous KI	8 months detectable, dead at 16	34%	Many	[135]
*Gcg* ^gfp/gfp^	2009	Alpha Cell Hyperplasia	*Gcg*	C57/BL6J	Homozygous KI	2 months	100%	None	[136]

Cre-LoxP-Mediated Transformations.

**Table 9:** Cre-LoxP GEMMs for MEN1 in pNENs. Many >5 other tumor types.

Name	Year	pNEN Subtype	Target Gene	Strain	Model Type	Tumor Development	% of Mice w pNENs	Other Tumors	References
*Men1*^TSM/+^, *Men1*^ΔN3–8/+^	2001	Pancreatic Islet Tumors	* MEN1 *	C57BL/6J × NIH Black Swiss females or 129/SvEvTacFBR	Cre-LoxP Heterozygous KO	9 months (hyperplasia)	28%	Many	[117]
*Men1* ^+/T^	2003	Insulinoma, Glucagonoma	*MEN1*	(129/Ola × 129/Sv)	Cre-LoxP Heterozygous KO	8+ months	>60%	Many	[148]
*Men1^loxP/loxP^ Rip-cre* ^+^	2004	Insulinoma	*MEN1*	C57BL/6J	Cre-LoxP Homozygous KO	4 months (hyperplasia), 9 months tumors	12/12 hyperplasia, 7/12 tumors	Pituitary, Prolactinomas	[140]
*MEN1*^+/−^;*Rb1*^ΔX2/+^	2007	Insulinoma, Glucagonoma	*MEN1, Rb1*	C57/129	Cre-LoxP Heterozygous KO	210–360 Days	10/18 hyperplasia, 1/18	Many	[118]
Pdx1-Cre, MEN1 ^f/f^,Pdx1-Cre MEN1^f/+^	2009	Insulinoma	*MEN1* (PDX1)	FVB;129Sv	Cre-LoxP Homozygous KO	5–6 months hyperplasia, 10–12 tumors	>80%	None	[141]
*MEN1*^F/F^-*GluCre^+^*	2010	Glucagonoma, Insulinoma, Mixed	*MEN1* (alpha cell only)	*R26R*	Cre-LoxP Homozygous KO	2–3 months (hyperplasia), 7 months	100%	None	[142]
Glu-Cre;Men1 f/+	2010	Glucagonoma, Insulinoma	*MEN1* (alpha cell only)	Glu-Cre;Z/AP	Cre-LoxP Homozygous KO	13–14 months	100%	None	[143]
βMen1/Bcat-KO	2014	Insulinoma	*MEN1*, β-Catenin	129/SvJ × C57BL/6J	Cre-LoxP Homozygous KO	8 months	33%	None	[144]
(*Men1^L/L^*/*RIP2-CreER*)	2017	Insulinoma	*MEN1*	C57Bl/6 × 129S	Cre-LoxP Homozygous KO	2–3 months	100%	None	[145]
MPR (*Men1^flox/flox^Pten^flox/flox^* RIP-Cre)	2020	PNETG1/G2 (WD)	*MEN1, PTEN*	Mixed	Cre-LoxP Homozygous KO	7 weeks	100%	Pituitary	[146]
(*Men1^flox/flox^ Pten^flox/flox^* MIP-Cre)	2020	PNETG1/G2 (WD)	*MEN1, PTEN*	Mixed	Cre-LoxP Homozygous KO	7 weeks	100%	Pituitary	[146]
* GFAP*^Δ*Men1*^, *GFAP*^Δ*Men1*^, *Ss*^−/−^, *GFAP*^Δ*Men1*;Δ*Kif3a*^; *Sst*^−/−^	2022	Islet Hyperplasia	*MEN1,* Sst, Kif3a (GFAP Expressing)	C57BL/6J	Cre-LoxP Homozygous KO	15–24 months	50%	Pituitary Prolactinomas, Gastric	[147]
* Sox10* ^Δ*Men1*^	2022	Islet Hyperplasia	*MEN1* (Sox10 expressing cells)	C57BL/6J	Cre-LoxP Transgenic, Induced Homozygous KO	10–12 months	57%	Gastric	[147]

**Table 10:** Other Cre-LoxP pNEN GEMMs. Many >5 other tumor types. NA = not available.

Study Name	Year	pNEN Subtype	Target Gene	Strain	Model Type	Tumor Development	% of Mice w pNENs	Other Tumors	References
Pdx1-Cre, VHL^f/f^	2009	Adenomas (VHL)	*VHL* (PDX1)	A/J and C57BL/6	Cre-LoxP Homozygous KO	16–18 months	NA	Pancreatic	[149]
RenCre x floxed p53/Rb1	2014	Glucagonoma	*p53, Rb*	C57BL/6J	Cre-LoxP Homozygous KO	22 weeks	100%	Sarcoma	[150]
*Rosa-CreER; FN ^f/f^ ; RIP-Tag*	2015	Islet Cell Tumors	* Fibronectin *	Many	Cre-LoxP Homozygous KO, Transgenic	7–11 weeks	100%	None	[151]
* Pdx1-tTA; tet-o-MT; p48-cre p16/p19^lox/lox^*	2016	pNET (Beta Cell Hyperplasia)	*PyMT*, *INK4A/ARF *(*PDX1*, *PTF1A*)	ICR, C57BL/6, FVB/N	Cre-LoxP Transgenic	400–600 days (survival)	3/35 (8.6%)	Pancreatic Acinar Ductal Carcinoma	[152]
* RIP7-rtTA; tet-o-MT; p48-cre p53^lox/lo^*	2016	pNET (Beta Cell Hyperplasia	*PyMT*, *p53 *(*RIP*, *PTF1A*	ICR, C57BL/6, FVB/N	Cre-LoxP Transgenic	400–600 days (survival)	2/12 (16.7%)	None	[152]
* RIP7-rtTA; tet-o-MT; p48-cre p16/p19^lox/lox^*	2016	pNET (Beta Cell Hyperplasia	*PyMT*, *INK4A/ARF* (*RIP*, *PTF1A*)	ICR, C57BL/6, FVB/N	Cre-LoxP Transgenic	400–600 days (survival)	12/60 (20%)	None	[152]
* RIP7-rtTA; tet-o-MT; p48-cre p53^lox/lox^; p16/p19^lox/lox^*	2016	pNET (Beta Cell Hyperplasia	*PyMT*, *p53*(*PDX1*, *PTF1A*	ICR, C57BL/6, FVB/N	Cre-LoxP Transgenic	400–600 days (survival)	12/30 (40%)	None	[152]
*Pdx1-Cre;Rb ^f/^*	2020	pNET (WD)	*Rb* (PDX1)	*Pdx1-Cre*, *Rosa26R*, *Rb flox*	Cre-LoxP Homozygous KO	18–20 months	80%	None	[153]
*Pdx1-Cre;Trp53^R172H^;Rb ^f/f^*	2020	pNET	*p53, Rb* (PDX1)	*Pdx1-Cre*, *Rosa26R*, *Rb flox*, *LSL-Trp53^R172H^*	Cre-LoxP Homozygous and Heterozygous KO	6 months	100%	None	[153]

**Table 11:** Genetic Non-Mouse Models of pNENs. NA = not available.

Study Name	Year	Host Species	Subspecies Model	Tumor Type	Gene	Establishment Method	Tumor Development	Success Rate	References
Chick	1977	Rat	NEDH inbred albino	Insulinoma	NA	X-ray-Induced	4 months	92%	[61]
Maisello	1984	Rat	Wistar	Islet Cell Tumor	NA	Chemical	11–24 months	81–100%	[154]
a/b-SV40 Tag	1994	Rat	Transgenic- Sprague Dawley	Islet Cell Tumors	a/b-SV40 Tag	Transgenic	3–5 months	33%	[155]
PEPCK-TAg	1999	Rat	Transgenic Sprague-Dawley	Islet Cell Carcinomas	SV40 Tag (PEPCK Promoter)	Transgenic	5–8 months	100%	[156]
pPEPCK-TGFAlpha	1999	Rat	Transgenic Sprague-Dawley	Islet Cell Carcinomas	TGFAlpha (PEPCK Promoter)	Transgenic	5–8 months	100%	[156]
*z-myod*–MYCN, core-z-myod-MYCN	2004	Zebrafish	NA	pNET	* MYCN *	Transgenic	4–6 months	4/250 (1.6%)	[159]
* rb1/rbl1 *	2020	Frog	* X. tropicalis *	SC pNEC	* Rb1, rbl1 *	CRISPR/Cas9 Homozygous KO	70 days	86%	[161]
* rb1/rbl1/tp53^cr2^*	2020	Frog	* X. tropicalis *	SC pNEC	* Rb1, rbl1, tp53 *	CRISPR/Cas9 Homozygous KO	70 days	77%	[161]

**Table 12:** Three-dimensional cell models of pancreatic neuroendocrine tumors, including spheroids, organoids, and bioreactor models. IN—insulinoma, NF—non-functioning, LC—large cell, IHC—immunohistochemistry. NA = not available.

Model Type	Cell Source	Success Rate	Year	Studies Performed	References
Spheroid	BON-1, QGP-1	100%	2012, 2014	Formation, Drug Screen, IHC, Model Comparison	[164,165]
Spheroid	BON-1	NA	2018	Formation, Radiation, IHC	[166]
Spheroid	BON-11	100%	2019	Formation, Drug Screen, IHC	[167]
Spheroid	BON-1	NA	2019	Formation, Drug Screen	[168]
Spheroid	BON-1, HMEG2725	NA	2020	Formation, Drug Screen	[169]
Spheroid	BON-1, QGP-1	NA	2020	Drug Screen	[170]
Spheroid	BON-1, QGP-1	NA	2022	Formation, Drug Screen	[171]
Spheroid	BON-1, INS-1E, NT-3, Primary pNET	4/4 (100%) (Primary Drug Screen)	2022	Formation, IHC, Drug Screen	[172]
Organoid	pNETG3, pNEC	3/8 (37.5%)	2020	IHC, Genomic, RNAseq, Methylation Array, Drug Screen, PDX Formation, Transcriptomic, CRISPR/Cas9	[82]
Organoid	pNET (INS, NF)	8/11 (72.7%) Culture 6/7 (86%) Drug Testing	2021	IHC, Drug Screen	[173]
Organoid	pNETG1, G2, pNEC	4/39 (10.3%)	2022	IHC, Genomic, Transcriptomic, Drug Screen	[174]
Organoid	pNET	4/13 (30.7%)	2022	IHC, Media Comparison	[175]
Organoid	pNET, pNEC (LC)	pNET 0%, pNEC 1/1 (100%)	2022	IHC, Drug Screen, Genomic, RNAseq	[176]
Organoid, Spheroid	pNETG3, pNEC (LC), QGP-1, NT-3	3/3 (100%)	2022	IHC, Drug Screen, Genomic, RNAseq, Transcriptomic, Model Comparison	[177]
Bioreactor	BON-1, QGP-1, PDX, Primary pNET	100% (Both)	2020, 2021	IHC, Drug Screen, Flow Cytometry, Invasion	[178,179]
